# High-Throughput Chemical Screens Identify Disulfiram as an Inhibitor of Human Glioblastoma Stem Cells

**DOI:** 10.18632/oncotarget.707

**Published:** 2012-10-23

**Authors:** Parvinder Hothi, Timothy J. Martins, LiPing Chen, Loic Deleyrolle, Jae-Geun Yoon, Brent Reynolds, Greg Foltz

**Affiliations:** ^1^ The Ben and Catherine Ivy Center for Advanced Brain Tumor Treatment, Swedish Neuroscience Institute, Seattle, WA, USA; ^2^ Quellos High-throughput Screening Core, UW Medicine, Seattle, WA, USA; ^3^ McKnight Brain Institute, University of Florida, Gainesville, FL, USA

**Keywords:** disulfiram, glioblastoma, high-throughput chemical screens, stem cells

## Abstract

Glioblastoma Multiforme (GBM) continues to have a poor patient prognosis despite optimal standard of care. Glioma stem cells (GSCs) have been implicated as the presumed cause of tumor recurrence and resistance to therapy. With this in mind, we screened a diverse chemical library of 2,000 compounds to identify therapeutic agents that inhibit GSC proliferation and therefore have the potential to extend patient survival. High-throughput screens (HTS) identified 78 compounds that repeatedly inhibited cellular proliferation, of which 47 are clinically approved for other indications and 31 are experimental drugs. Several compounds (such as digitoxin, deguelin, patulin and phenethyl caffeate) exhibited high cytotoxicity, with half maximal inhibitory concentrations (IC_50_) in the low nanomolar range. In particular, the FDA approved drug for the treatment of alcoholism, disulfiram (DSF), was significantly potent across multiple patient samples (IC_50_ of 31.1 nM). The activity of DSF was potentiated by copper (Cu), which markedly increased GSC death. DSF–Cu inhibited the chymotrypsin-like proteasomal activity in cultured GSCs, consistent with inactivation of the ubiquitin-proteasome pathway and the subsequent induction of tumor cell death. Given that DSF is a relatively non-toxic drug that can penetrate the blood-brain barrier, we suggest that DSF should be tested (as either a monotherapy or as an adjuvant) in pre-clinical models of human GBM. Data also support targeting of the ubiquitin-proteasome pathway as a therapeutic approach in the treatment of GBM.

## INTRODUCTION

Glioblastoma multiforme (GBM), the most common primary brain tumor in adults, remains an incurable and rapidly fatal disease [1]. After primary therapy, consisting of surgical resection followed by concurrent radiation and chemotherapy, the average time to recurrence is only 9 months and once the tumor recurs, average survival is limited to 6 months [2]. According to the hierarchical model of tumorigenesis, the presumed cause of tumor recurrence is the sub-population of tumor-initiating cells or glioma stem cells (GSCs) that persist after primary therapy [3]. Although the relationship between the GSC population and bulk tumor remains controversial, accumulating evidence supports the notion that GSCs have the ability to replicate (self-renewal) and generate all, or most, of the diverse cell types found within the mature recurrent tumor. The GSC population has been shown to be resistant to standard therapy, either because of distinct biophysical and genetic properties, or possibly due to migration outside of the treatment field [4, 5]. Recent reports in the literature support the importance of targeting cancer stem cells as an additional strategy to improve overall response to cancer therapy [6-10]. In GBM, targeting cancer stem cells as the potential cause of tumor recurrence clearly represents an opportunity to improve patient prognosis.

With this in mind, we performed high-throughput chemical screens (HTS) to identify compounds with the potential to prevent tumor recurrence by inhibiting GSC proliferation. We screened the MicroSource Spectrum collection; a 2,000 compound library consisting of FDA approved drugs, compounds that have reached late-phase clinical trials, experimental drugs, and natural products. This collection was selected based on the structural and biological diversity it provides for HTS. Furthermore, the identification of drug candidates that are already approved for another indication should have a rapid route into the clinic, whereas the identification of new agents provides a much needed platform for drug development. Because commercially available immortalized cancer cell lines do not account for the genetic diversity between individual patients or the cellular heterogeneity of the tumors from which they are derived, we used patient-specific cell cultures for HTS which contain heterogeneous tumor cell populations and, more importantly, preserve the GSC phenotype [11]. The inherent heterogeneity of GBM is reflected in GSCs, which differ in their proliferative potential, tumor-initiating ability and therapeutic responses, and more closely resemble the parent tumor both genotypically and phenotypically [12].

We report the half maximal inhibitory concentrations (IC_50_) for several drug candidates with the potential to inhibit GSC proliferation. From these we have identified disulfiram (tetraethylthiuram disulfide, Antabuse®, DSF), a clinically approved drug for the treatment of alcoholism [13], as a potent inhibitor of multiple patient-derived GSCs. We demonstrate that the anti-tumor effects of DSF are a result of proteasome inhibition and the subsequent induction of tumor cell death. In addition, we show that DSF activity is dependent on the presence of copper ions (Cu), consistent with the formation of a thiocarbamate-copper complex that functions as a proteasome inhibitor. Accumulating evidence suggests that proteasomal activity contributes to tumorigenesis by promoting tumor cell proliferation, down regulating apoptosis, and increasing angiogenesis. The ubiquitin-proteasome pathway is therefore considered an important target in cancer therapy [14]. Combined with the fact that proteasome inhibitors are emerging as promising therapeutic agents against glioma [15], treatment with a relatively nontoxic proteasome inhibitor such as DSF could be an attractive approach to test in clinical trials for GBM patients.

## MATERIALS AND METHODS

### Ethics Statement

This study was reviewed and approved by Western IRB (IRB00000533) in compliance with the ethical principles as set forth in the report of the National Commission for the Protection of Human Subjects of Biomedical and Behavioral Research entitled “Ethical Principles and Guidelines for the Protection of Human Subjects of Research (Belmont Report)”. The research protocol was also approved by the Swedish Neuroscience Institute research steering committee. All participants provided written informed consent according to IRB guidelines prior to participation in this study.

### Patient samples and cell lines

Tumors were obtained from surgeries performed at Swedish Medical Center (Seattle, WA) according to institutional guidelines. Patient samples used in this study were diagnosed as WHO grade IV glioblastoma multiforme (Table S1).

GSC cultures were established from freshly resected tumor tissues and maintained in NeuroCult® NSA medium (Stem Cell Technologies) with B-27 serum-free supplement (Invitrogen), 20 ng/mL epidermal growth factor (EGF) and 20 ng/mL fibroblast growth factor (FGF-2) as described [11]. In brief, tissue samples were minced into 1 mm^3^ fragments and digested with Accutase (Sigma) at 37 ^°^C for 15-20 minutes. NSA medium was added to quench Accutase activity and cell suspensions were passed through 70 μm nylon mesh. The suspensions were centrifuged at 1000 rpm for 5 minutes, resuspended in fresh NSA, and plated into T75 flasks pre-coated with laminin (1:100 in PBS; Sigma). To evaluate neurosphere formation ability of GSCs (Figure S1), single cell suspensions were plated directly into ultra low attachment dishes (Corning) and maintained in NSA medium.

Human neural stem cells (NSCs) and G144 GSCs were obtained from Celprogen (San Pedro, CA) and the BioRep Cell Repository (Milan, Italy), respectively. SKBR3 cells were purchased from American Type Culture Collection (ATCC, Manassas, VA) and grown in McCoy's 5A medium with 10% FBS. Commerically available cell lines were authenticated by the corresponding vendor by Short Tandem Repeat (STR) and used within six months. GSCs have not been authenticated due to lack of reference STR. With the exception of G144 (which was provided by the vendor at passage 22), cells were used at ≤ passage 5.

Bathocuproinedisulfonic acid (BCPS), copper (II) sulfate (CuSO_4_), dimethyl sulfoxide (DMSO) and tetraethylthiuram disulfide (disulfiram, DSF) were purchased from Sigma. Zinc gluconate was from MP Biomedicals.

### Immunocytochemistry

GSCs were grown on laminin coated glass chamber slides (Nunc) in NSA medium. Cells were fixed with 4% paraformaldehyde for 15 minutes at room temperature, treated with 5% goat serum (Invitrogen), and stained with antibodies as appropriate. For differentiation studies, cells were grown for 10 days in NSA medium either with or without EGF and FGF-2. Medium was replaced every 3 days. Primary human antibodies for nestin, vimentin, CD44, GFAP, TUJ-1 and O4 (1:50) were from R&D Systems. Goat secondary antibodies conjugated to Alexa dyes (1:500) were from Invitrogen. DAPI (Sigma) was used as the nuclear counterstain, and images were acquired using a Nikon Ti-U inverted fluorescence microscope liked to a DS-U2 camera.

### Compound library

The library used was the MicroSource Spectrum collection (MicroSource Discovery Inc.). A library of 2,000 compounds composed primarily of FDA approved compounds (50%), natural products (30%), and other bioactive components (20%). The compounds were supplied by the vendor as 10 mM solutions in DMSO at > 95% purity.

### High-throughput chemical screens with GSCs

Cells were added to laminin coated 384-well plates at a density of 800 cells per well using a Thermo Scientific Matrix WellMate, and incubated overnight to allow attachment. Compounds were added (0.01, 0.1, 1 and 10 μM) to GSCs using the CyBi-Well vario and incubated at 37 ^°^C for 96 hours. CellTiter-Glo (Promega) was added to individual wells and, following 20 minutes incubation on an orbital shaker, luminescence was measured on a Perkin Elmer EnVision. HTS was performed on five patient-derived cultures (SN143, SN175, SN179, SN186 and G144) in duplicate. Measurements were corrected for background luminescence. Percentage cell viability is reported as the mean and standard deviation (SD) relative to DMSO control.

### Dose response curves and IC_50_ calculations

The potency of active compounds was quantified by generating 8-point dose response curves. Experimental conditions were identical to those described for the initial screen. Dose response data were also obtained for NSCs, for the comparison of drug potency on normal versus malignant cells. Test compounds were added to cell lines at concentrations ranging from 0.005 – 10 μM. IC_50_ values were calculated by fitting data to the standard four-parameter sigmoidal dose response curve. Curve fitting was performed with GraFit software.

### ToxCount cell viability assays

Cell viability was measured using ToxCount assays per the manufacturer's recommendations (Active Motif). GSCs were plated in laminin coated 96-well plates at a density of 2,000 cells per well, allowed to attach overnight, and treated with either DMSO or DSF, ranging from 0.01 – 10 μM, for 96 hours. For investigating the effect of metal ions on cell viability, GSCs were treated with DSF combined with either copper sulfate or zinc gluconate (at a 1:1 molar ratio). Prior to taking proliferation measurements, ToxCount reagents were incubated with GSCs for 30 minutes at 37 ^°^C. The conversion of calcein AM to green fluorescent calcein in viable cells was measured using the IsoCyte (BlueShift Biotechnologies) with a 510/540 nm band pass filter. Percentage cell viability is reported as the mean and SD (of six replicate measurements) relative to DMSO control.

### Proteasomal chymotrypsin-like activity assays

The chymotrypsin (CT)-like activity was measured in GSCs using Proteasome-Glo assays per the manufacturer's recommendations (Promega). GSCs were plated in laminin coated 96-well plates at a density of 2,000 cells per well, allowed to attach overnight, and treated with DMSO, DSF or DSF–Cu (1 or 10 μM), for 96 hours at 37^°^C. After incubation, Proteasome-Glo Reagent containing the proteasome substrate for CT-like activity (Succinyl-leucine-leucine-valine-tyrosine-aminoluciferin) was added to each well. Plates were mixed on an orbital shaker for 2 minutes and then incubated at room temperature for 15 minutes. Luminescence was read on a Perkin Elmer EnVision and measurements were corrected for background luminescence. Percentage (CT)-like proteasomal activity is reported as the mean and SD (of six replicate measurements) relative to DMSO control.

### Aldefluor analysis and flow cytometry

Aldehyde dehydrogenase (ALDH) activity was determined using the Aldefluor assay per the manufacturer's instructions (Stem Cell Technologies). Cells (1 × 10^6^) were treated with either DMSO or DSF–Cu (1 μM for 24 hours) and resuspended in Aldefluor assay buffer containing the ALDH substrate, bodipy-aminoacetaldehyde (BAAA; 5 μM), for 45 minutes at 37 ^°^C. As a negative control for each treatment condition, cells were incubated with 15 μM diethylaminobenzaldehyde (DEAB), a specific ALDH inhibitor. Fluorescence activated cell sorting (FACS) was performed using a BD Influx. Aldefluor fluorescence was excited at 488 nm and emission was detected using a standard fluorescein isothiocyanate (FITC) 530/40 nm band-pass filter. The ALDH+ population was determined relative to the corresponding DEAB treated control. Data was acquired using Spigot 6.1.4 and analyzed with FlowJo 7.6.5.

### Xenograft experiments

Animal work was approved by the Institutional Animal Care and Use Committee (IACUC) at The University of Florida. To verify tumor initiating ability, non-obese diabetic/severe combined immunodeficient (NOD/SCID) female mice 8-10 weeks of age were anesthetized and received a flank subcutaneous injection of GSCs (1 × 10^6^) in 200 μl of medium and 100 μl of Matrigel (BD). The animals were divided into equal groups based on receipt of GSC line (n = 5), monitored for tumor growth, and euthanized once tumors reached ~2000 mm^3^ in size. Tumor volume is reported as the mean and standard error of the mean (SEM).

## RESULTS

### Initial characterization of patient-derived GSC cultures

A cancer stem cell is defined as an undifferentiated cell with the ability to self-renew, differentiate to multiple lineages, and initiate tumors that resemble the parent tumor [3]. CD133, A2B5 and SSEA-1 have been identified as potential GSC markers, however, the limits of using surface markers to isolate pure tumor-initiating populations of GBM are well recognized [16]. We therefore adopted a cell culture model that preserves the GSC population from human GBM samples [11], and has the ability to produce the large number of cells required for HTS.

GSCs were confirmed in patient-derived cultures by functional assays of self-renewal (serial neurosphere formation), differentiation potential, and tumor propagation *in vivo*. GSCs formed neurospheres, expressed neural stem cell markers (nestin, vimentin and CD44; consistent with previous reports of tumorigenic adherent GSCs [11]), and differentiated into cell lineages expressing makers for astrocytes (GFAP+), neurons (TUJ1+) and oligodendrocytes (O2+). Furthermore, tumors formed in NOD/SCID mice approximately 6-8 weeks post injection confirming the presence of the GSC phenotype (Figure S1).

### High-throughput chemical screens of 2,000 compounds against patient-derived GSCs

The Spectrum collection was initially screened against five GSC cultures (SN143, SN175, SN179, SN186, and the commercially available G144) to identify inhibitors of GSC proliferation. 1867 (93.4%) of the compounds showed less than 50% cell death and were not pursued further. Although several of these compounds were cytotoxic at 10 μM, cell viability was 100% at the lower concentration range (0.1 – 1 μM) indicating a lack of concentration dependence. The remaining 133 compounds (6.6%) showed some cytotoxicity at both 1 and 10 μM. From these positive hits, 78 compounds repeatedly inhibited GSC growth across the five samples tested (Table S2). The remaining 55 compounds were not common inhibitors but did inhibit the proliferation of specific patient-derived GSCs (Table S3). The compounds identified represent multiple classes of drugs and natural products, including antineoplastics, cardiotonics, antihelminthics, and others, as indicated in Table 1. The total number of active agents, for each class of drug per GSC line, was determined (Table 1). The common GSC inhibitors were further evaluated for potency.

**Table 1:** Pharmacological classes for inhibitors of GSC proliferation

Class^a^	Total in class	Active agents					% Active in class
		SN143	SN175	SN179	SN186	G144	
Alcohol antagonist	3	1	1	2	1	1	66.7
Antihelminthic	33	7	8	8	7	8	24.2
Antiarrhythmic	24	0	1	2	1	1	8.3
Antibacterial	227	11	12	11	11	10	5.3
Antifungal	55	5	4	6	5	5	10.9
Antineoplastic	115	29	31	29	28	27	27.0
Antihyperlipidemic	12	3	3	5	4	3	41.7
Antihypertensive	63	1	3	2	1	0	4.8
Anti-infective	11	2	2	3	3	2	27.3
Antipsychotic	22	1	1	1	1	1	4.5
Cardiotonic	14	10	10	10	10	10	71.4
Diuretic	16	0	0	1	0	0	6.3
H1 antihistamine	11	1	1	1	0	1	9.1
Immunosuppressant	5	1	2	1	0	0	40.0
Psychotropic	9	0	3	1	0	0	33.3
Sclerosing agent	2	1	1	1	1	1	50.0
Vasodilator	35	0	0	2	2	1	5.7
Undetermined activity	444	19	22	21	17	17	4.95
Total agents	1101^b^	92	105	107	92	88	9.72^c^

a Only classes containing inhibitors of GSC proliferation are shown.

b The remaining 899 agents constitute other non-active pharmacological classes.

c Percent active in the complete screen (2,000 compounds) is 5.35%.

### Half maximal inhibitory concentrations of inhibitors of GSC proliferation

Dose response curves were generated for compounds identified from the initial screen to determine the half maximal inhibitory concentration (IC_50_) for each compound and GSC line (Table S4). It has recently been reported that commonly used chemotherapy agents (cisplatin and TMZ) inhibit NSC rather than GSC proliferation *in vitro* [17], and we observed similar effects with several compounds (*e.g*. amsacrine, camptothecin, deguelin, salinomycin; Table S4). Taking the effects of TMZ on cultured NSCs into consideration, we chose not to evaluate compounds based on their toxicity for normal versus malignant cells. The IC_50_ values for GSCs varied considerably from low nanomolar to high micromolar, and in some cases, for the same drug across the different patient samples (*e.g*. 0.28 – 5.62 μM and 0.39 – 18.82 μM for etoposide and fluvastatin, respectively). Results presented herein confirm the heterogeneity of response to drugs between patients.

**Figure 1:**
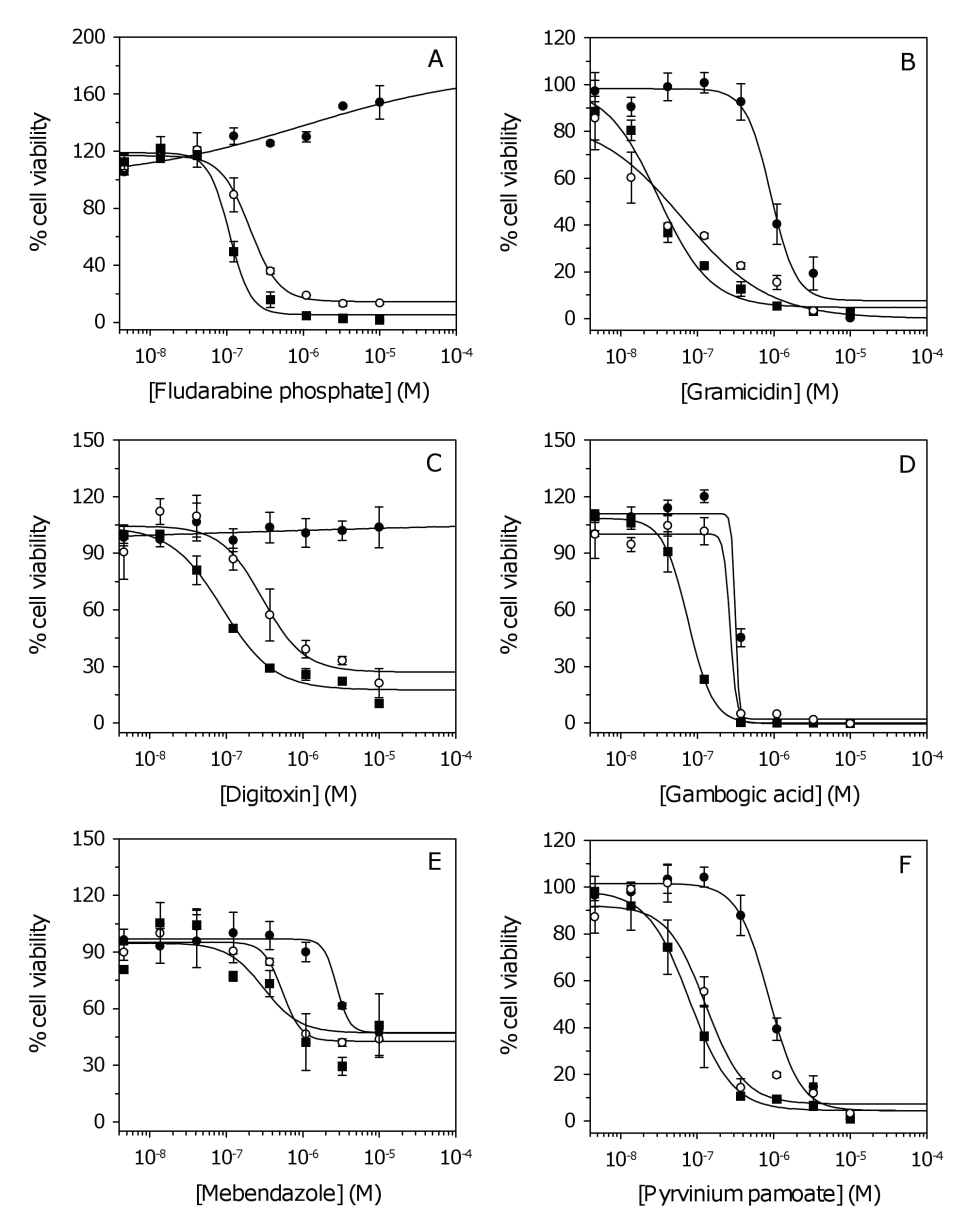
Dose response curves for inhibitors of GSC proliferation (A-D) Examples of dose response data for antineoplastics, antibacterials, cardiotonics, and compounds with undetermined activity, respectively. (E-F) Dose response curves for the antihelminthics mebendazole and pyrvinium pamoate, respectively. For clarity, three (of six) cell lines are shown corresponding to NSCs and GSCs with the lowest and highest IC_50_. Data represent the mean and SD from two independent experiments. Data were fit to the standard sigmoidal dose response curve. Closed circles, NSCs; open circles, GSC with lowest IC_50_; closed squares, GSC with highest IC_50_ (Table S4).

**Figure 2:**
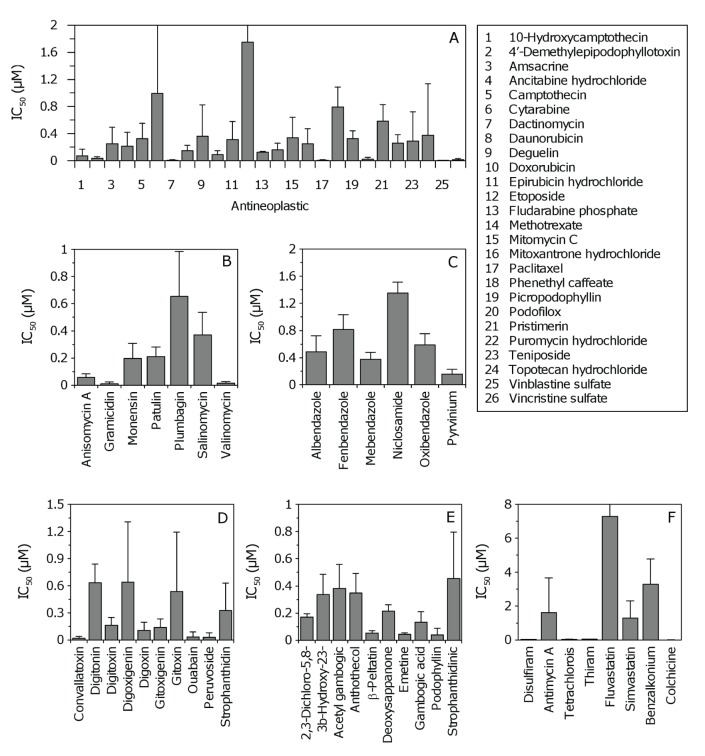
Average half maximal inhibitory concentrations (IC_50_) for inhibitors of GSC proliferation (A-E) Data for antineoplastics, antibacterials, antihelminthics, cardiotonics, and compounds with undetermined activity, respectively. Error bars correspond to SD (*i.e.* IC_50_ variation across five GSC lines). SD for cytarabine and etoposide are 1.4 and 2.2, respectively (not shown for clarity). For panel E, abbreviated compounds are 2,3-dichloro-5,8-dihydroxynapthoquinone, 3β-hydroxy-23,24-bisnorchol-5-enic acid, deoxysappanone B 7,4'-dimethyl ether, and strophanthidinic acid lactone acetate, respectively. (F) Data for compounds constituting other pharmacological classes (alcohol antagonist, antifungal, antihyperlipidermic, anti-infective and antigout agent). SD for fluvastatin is 8.8 (not shown for clarity). Abbreviated compounds are tetrachloroisophthalonitrile and benzalkoium chloride.

Examples of dose response curves for each pharmacological class are shown in Figure 1A-F. Due to the large variation observed across patient samples (Table S4), average IC_50_ values where determined to evaluate the general potency of individual compounds against GSCs (Figure 2). The average IC_50_ for antineoplastics varied considerably from 0.004 – 1.749 μM for vinblastine and etoposide, respectively (Table S4). Antibacterials, antihelminthics, cardiotonics, and compounds with undetermined activity had IC_50_ values that were generally less than 1 μM (Figure 2B-E, respectively). The other pharmacological classes (alcohol antagonist, antifungals, antihyperlipidermics, anti-infective and antigout agent) showed large variation in potency (Figure 2F, Table S4).

As a crude estimate of which candidates were most likely to be active in the central nervous system (CNS), we compared their drug-like properties according to Lipinski's Rule of 5 and molecular polar surface area (PSA), predictors of orally active drugs [17] and blood-brain barrier (BBB) penetration [18], respectively. With the exception of some pharmacological classes, such as antibacterials and cardiotonics, the Rule of 5 states that most drug-like molecules have a molecular weight of 500 kDa or less, an octanol/water partition coefficient (log*P*) of 5 or less, up to 5 hydrogen bond donors (OH + NH count), and up to 10 hydrogen bond acceptors (O + N atoms) [18]. Compounds with a PSA of less than 70 Å^2^ usually have a higher probability of penetrating the BBB [19].

Several clinically approved drugs (*e.g*. antibacterials, dactinomycin and vincristine) are known to have poor BBB permeability (Table S5). The CNS-active antihelminthic, mebendazole, is cytotoxic to GSCs (Figure 1E) and has recently been shown to be active in GBM xenografts [20]. We identified a more potent antihelminthic, pyrvinium pamoate (Figure 1F); however, this drug is poorly absorbed in humans and therefore unlikely to have a significant effect *in vivo* [21]. The cardiotonics or cardiac glycosides represent the pharmacological class most active against GSCs (Table 1, Figure 1C). The ability of digitalis to inhibit the proliferation of malignant cells has been established for some time, but its use in cancer treatment has been widely debated due to concerns of serious adverse effects in humans [22]. Of the remaining clinically approved drugs, disulfiram (DSF), which is currently used for alcohol aversion therapy [13] was identified as a potent inhibitor of GSC proliferation. Several experimental compounds identified from the Spectrum collection (*e.g*. deguelin, patulin and phenethyl caffeate) may have the potential to reduce tumor growth *in vivo* (PSA ≤ 70 Å^2^; Table S5). However, many newly identified drugs tested in research or pre-clinical settings are common apoptosis inducers with unclear mechanisms of action (*e.g*. gambogic acid [22]; Figure 1D), which limits their transition from research to clinical applications. The advantages of a clinically approved drug, such as DSF, are several-fold since the pharmokinetics are already well-established. Furthermore, it is known that DSF is rapidly reduced in the bloodstream to an active metabolite, diethyldithiocarbamate (DTTC) or methyl diethylcarbamodithioate (Me-DTTC), which can readily penetrate the BBB (PSA = 35.22 and 60.63 Å^2^, respectively) [13]. Data therefore suggest that DSF is a lead candidate for further studies.

### DSF–Cu inhibits the proliferation of GSCs derived from multiple GBM patients by inhibiting proteasome activity

**Figure 3:**
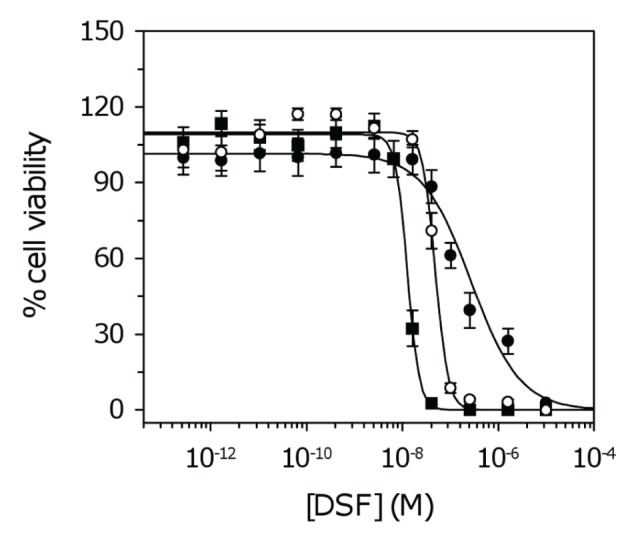
Dose response curves for GSCs treated with DSF For clarity, three of ten cell lines are shown corresponding to NSCs and GSCs with the lowest and highest IC_50_. Data represent the mean and SD from four independent experiments. Data were fit to the standard sigmoidal dose response curve. Closed circles, NSCs; open circles, highest IC_50_ (SN195); closed squares, lowest IC_50_ (SN235) (Table S6).

Our *in vitro* data suggest DSF markedly inhibits the proliferation of patient-derived GSCs (average IC_50_ of 34.1 ± 6 nM). Given the inherent heterogeneity of GBM, and the large variation in IC_50_ values observed for several other compounds (Table S4), we tested additional patient samples. IC_50_ values remained in the low nanomolar range (12.1 – 56.3 nM; average 31.1 ± 12.9 nM) across the total patient population (Figure 3, Table S6).

**Figure 4:**
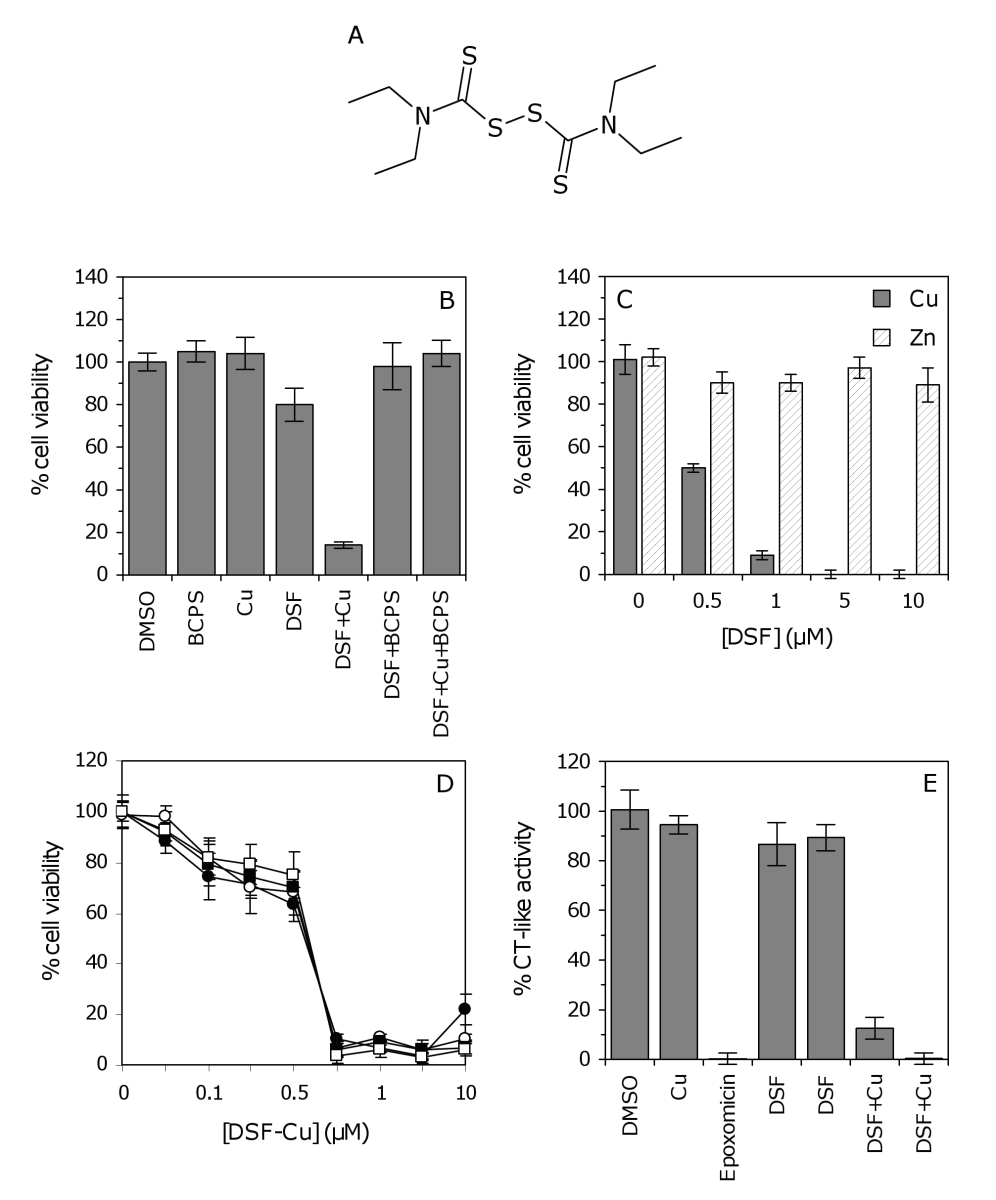
The effect of the DSF–Cu complex on GSC proteasomal activity (A) The chemical structure of DSF. (B) The anti-proliferative effect of DSF–Cu on GSCs. SN186 cells were treated with BCPS (100 μM), Cu^2+^ (10 μM), DSF (1 μM) or DSF–Cu (1 μM) for 96 hours, followed by ToxCount cell viability assays. (C) The anti-proliferative effect of DSF–Cu versus DSF–Zn. SN186 cells were treated with various concentrations of DSF–Cu or DSF–Zn (1:1 molar ratio), ranging from 0 – 10 μM, for 96 hours. Neither Cu nor Zn alone (10 μM) affected cell viability (presented as zero DSF). (D) Time course of GSC growth inhibition by the DSF–Cu complex. SN186 cells were treated with various concentrations of DSF–Cu (0 – 10 μM). Cell viability was measured after 24, 48, 72 or 96 hours (closed circles, open circles, closed squares and open squares, respectively). The inhibition of GSC proliferation was identical at 24 hours versus 96 hours, and the greatest degree of inhibition was observed at concentrations of 0.75 μM or higher. (E) Inhibition of the chymotrypsin (CT)-like proteasomal activity by DSF–Cu. SN186 GSCs were treated with DMSO, Cu (10 μM), epoxomicin (10 μM), DSF (1 μM), DSF (10 μM), DSF–Cu (1 μM) or DSF–Cu (10 μM), respectively, for 96 hours. CT-like activity was measured using Proteasome-Glo luminescent assays. For Panels B, C and E, columns and error bars represent the mean and SD of six replicate measurements, respectively.

DSF (Figure 4A) is a member of the dithiocarbamate family, compounds that have the ability to complex metal ions and form a proteasomal inhibitory complex that induces apoptosis [24]. To explore this mechanism of action in GSCs, cell viability was measured after treatment with either DSF or a 1:1 molar ratio of DSF and copper (Cu). Growth inhibition was significantly enhanced in GSCs treated with DSF–Cu (Figure 4B). The inhibitory effect observed in the absence of added copper (Figure 3) is therefore attributed to the presence of endogenous copper, which is generally added to media to promote cell survival. To confirm this, GSCs were cultured with DSF and the Cu^2+^ chelator bathocuproinedisulfonic acid (BCPS). As anticipated, the addition of BCPS reversed the anti-proliferative effect of DSF–Cu, and more importantly, reversed the inhibitory activity of DSF alone (Figure 4B). The inhibition of GSC proliferation was specific to DSF–Cu as cells treated with DSF and zinc (Zn) were not inhibited (Figure 4C). The DSF–Cu complex was highly potent above 0.75 μM and time course experiments revealed similar inhibitory effects at 24 versus 96 hours (Figure 4D).

It has been reported that inhibition of chymotrypsin (CT)-like proteasomal activity is associated with the induction of apoptosis in tumor cells [25]. To further evaluate the hypothesis that DSF inhibits proteasome function in GSCs, cells were assayed directly for CT-like activity. As a positive control cells were treated with epoxomicin (10 μM), a potent inhibitor of CT-like proteasomal activity [26]. DSF alone (1 or 10 μM) inhibited CT-like activity by ~10%, which can be attributed to the activity of DSF complexed with endogenous copper. The CT-like activity was significantly inhibited (by 88%) in GSCs treated with 1 μM DSF–Cu and completely abolished with 10 μM DSF–Cu (Figure 4E). Interestingly, treatment with DSF–Cu inhibited proteasomal activity to the same degree as epoxomicin (Figure 4E), confirming that DSF–Cu is a potent inhibitor of CT-like proteasomal activity. Taken together, results suggest that inhibition of GSC proliferation by DSF is dependent on the formation of a thiocarbamate-copper complex, which functions as an inhibitor of the CT-like proteasomal activity in GSCs and induces tumor cell death.

### The effect of DSF–Cu on aldehyde dehydrogenase activity in GSCs

**Figure 5:**
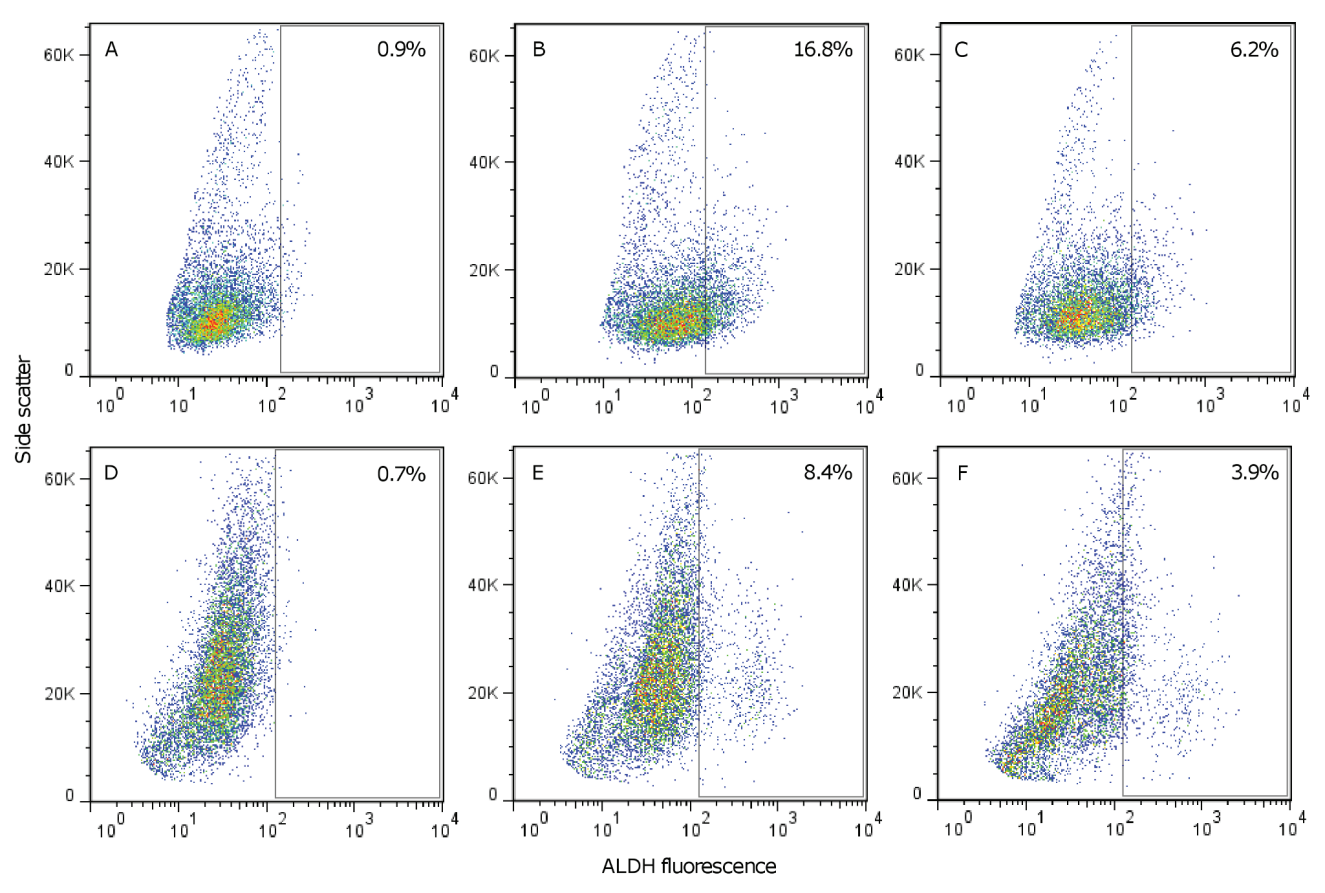
The effect of the DSF–Cu complex on ALDH activity (A-B) SKBR3 cells incubated with or without the ALDH inhibitor DEAB, respectively. An ALDH+ population of 16.8% (panel B) was determined relative to the DEAB control (panel A). (C) SKBR3 cells treated with 1 μM DSF–Cu for 24 hours. The ALDH+ population decreased to 6.2% after DSF–Cu treatment. (D-E) SN186 GSCs incubated with or without DEAB, respectively. An ALDH+ population of 8.4% was detected relative to DEAB. (F) SN186 GSCs treated with 1 μM DSF–Cu for 24 hours. The ALDH+ population was 3.9% following DSF–Cu treatment. The percentages shown in panels B, C, E and F correspond to the ALDH+ population determined after subtraction of control reactions.

Aldehyde dehydrogenase (ALDH) is believed to be a functional marker of cancer stem cells and involved in maintaining the progenitor cell phenotype [27, 28]. As DSF is an irreversible inhibitor of ALDH [29], we tested whether DSF–Cu treatment inhibited ALDH activity in GSCs. ALDH was quantified by flow cytometry using Aldefluor; a fluorescent reagent used to detect ALDH in human cells. SKBR3 breast cancer cells, which are known to express ALDH, were used as a positive control [30]. An ALDH+ population of 16.8% was detected in DMSO treated SKBR3 cells (Figure 5B), which was diminished in cells that had been treated with the ALDH inhibitor DEAB (Figure 5A). Treatment of SKBR3 cells with DSF–Cu (1 μM for 24 hours) reduced the ALDH+ population to 6.2% (Figure 5C). In DMSO treated GSCs, an ALDH+ population of 8.4% was detected relative to the matched DEAB treated sample (Figure 5D, E), which was reduced to 3.9% in DSF–Cu treated cells (Figure 5F). Although data obtained here suggest that DSF–Cu may inhibit ALDH positive populations, further studies are needed (across a range of patient samples) to establish the extent of ALDH activity in GBM, and to evaluate the contribution of ALDH inhibition to GSC death. The value of ALDH as a universal cancer stem cell marker remains controversial as some evidence suggests that high ALDH activity does not correlate with increased tumor initiating capacity [31]. It should also be noted that several isoforms of ALDH exist, different isoforms have been identified in stem cell populations [32], and there is ongoing debate as to which isoforms are detected by the Aldefluor assay [32, 33].

Another potent ALDH inhibitor, chloramphenicol [34], showed negligible activity against GSCs during the initial HTS (Figure S2). This suggests that chloramphenicol is not a general treatment option for GBM patients as hypothesized by others [35].

## DISCUSSION

GBM is associated with one of the worst 5–year survival rates of all human cancers, with an average survival time after diagnosis of only 12–14 months. Long-term survivors (defined as patients that survive for more than 36 months) represent only 3–5 % of the total patient population [36]. With the completion of large-scale genomic sequencing projects [37] and the development of improved transgenic mouse models [38] there has been considerable progress in the identification of genes and pathways which represent potential drug targets in GBM [39-44]. Here, we report a complementary approach using an unbiased HTS strategy for the screening of large chemical libraries and the identification of compounds active against patient-derived GSCs (Tables 1, S2 and S3). Our goal was to identify therapeutic agents with the potential to target the stem cell population, prevent tumor recurrence, and subsequently extend survival in GBM patients. In particular, we sought to identify agents already approved by regulatory agencies for human use, since existing drugs have well-established pharmokinetics and can be rapidly tested in phase II clinical trials. Dose response curves (Figure 1, Table S4) and average IC_50_ values (Figure 2) were used to evaluate drug potency. Of the multiple candidates identified, we chose to pursue the clinically approved drug DSF; a relatively non-toxic compound that can readily penetrate the BBB [13].

Although the anti-cancer properties of DSF have been known for over 40 years [45], very little is known about the activity of this drug on brain tumors. Our *in vitro* data clearly show that DSF can potently inhibit GSC proliferation across a range of patient samples (average IC_50_ = 31.1 nM; Figure 3, Table S6). The activities of dithiocarbamates have been attributed to their ability to form active complexes with metals (in particular copper) which have proteasomal inhibitory and apoptosis inducing capabilities [24]. This mechanism of action has been demonstrated for DSF in human breast, colon and prostate cancers [46, 47]. To explore this mechanism in GBM, we compared cell viability and proteasomal CT-like activity in GSCs following treatment with DSF or DSF–Cu. The anti-proliferative activity of DSF was dependent on Cu, and reversed in the presence of the Cu^2+^ chelator BCPS (Figure 4B). The inhibition of proteasomal CT-like activity was also potentiated by Cu (Figure 4E) and comparable to percentage cell survival (Figure 4B), confirming that DSF–Cu inhibits proteasome activity and induces cell death in cultured GSCs. We also observed that DSF–Cu has the ability to inhibit ALDH activity *in vitro* (Figure 5). ALDH promotes cell survival by protecting DNA from genotoxic damage and providing resistance to a wide range of anti-cancer drugs [28, 48]. The inhibition of ALDH is therefore considered an attractive approach for sensitizing resistant cell populations to the cytotoxic effects of chemotherapy agents [32, 33, 49].

Combined treatment with DSF and zinc gluconate has been reported to have induced >50% reduction in hepatic metastases and produced clinical remission in a patient with stage IV metastatic ocular melanoma [50]. With this in mind, we compared the anti-tumor activity of DSF–Zn relative to DSF–Cu. GSC proliferation was not affected by DSF–Zn (Figure 4C), suggesting that the DSF–Cu complex specifically inhibits proteasome activity in GSCs. It is generally believed that cancer cells and tissues, which contain elevated levels of copper and are dependent on proteasome activity for their survival, should be more sensitive to treatment with proteasome inhibitors than normal cells and tissues [14]. Selectivity of the DSF–Cu complex for malignant cells has been reported by others [51], and we also observed greater selectivity for GSCs than NSCs (IC_50_ 31.1 nM versus 282.5 nM, respectively). As many other anti-cancer agents are unable to distinguish between malignant and normal cell populations (which contributes to their toxicity *in vivo*), proteasome inhibitors are considered to be promising therapeutic agents for the treatment of cancer patients [14].

There are several proposed mechanisms by which proteasome inhibitors mediate anti-cancer effects. One mechanism is inhibition of the transcription factor Nuclear Factor kappa B (NF-kB), which is critical in tumorigenesis and over active in GBM [52]. NF-kB supports disease progression by increasing the proliferation of tumor cells, inducing the transcription of anti-apoptotic genes as well as genes involved in DNA damage responses, and by promoting angiogenesis [14]. Inhibition of proteasomal activity promotes accumulation of the NF-kB inhibitor, I-kB, which prevents NF-kB activation and subsequently down regulates factors related to cancer progression [14]. DSF, its monomer DDTC, and their derivatives are widely recognized as inhibitors of the NF-kB pathway [53, 54], and it is well-established that NF-kB inhibition sensitizes tumor cells to commonly used chemotherapeutic agents [55]. Proteasome inhibitors (including DSF) are therefore known for their ability to potentiate the activity of other anti-cancer drugs [55, 56]. DSF has the added capability to decrease multidrug resistance by inhibiting the P-glycoprotein extrusion pump [57], further supporting its use as an adjuvant in cancer therapy.

DSF has also been reported to increase intracellular oxidative stress and induce apoptosis in melanoma cells [58], as well as inhibit angiogenesis and significantly reduce Lewis lung metastatic growth and C6 glioma development *in vivo* [59]. Another study has suggested that DSF is a DNA methyltransferase inhibitor, capable of demethylating gene promoters and reactivating the expression of epigenetically silenced genes in prostate cancer [60]. Consistent with the multiple effects of DSF, many studies have shown that the underlying mechanism of action of the proteasome inhibitor bortezomib is complex, and that the drug affects a range of cellular functions [14].

Studies with breast cancer xenografts suggest that DSF reduces tumor progression *in vivo* [46, 61]. High levels of copper have been reported in patients with brain tumors [62], and DSF is systemically absorbed as its bis(diethyldithiocarbamato) copper complex after passage through the acidic environment of the stomach [13], which implies that the DSF–Cu complex can be readily formed *in vivo*. It is also important to note that DSF has been used in the clinic long-term with generally only mild side effects [63]. Furthermore, DSF has been administered (at 500 mg/daily for 3 months followed by 250 mg/daily for another 50 months) to produce clinical remission in a 64 year old female with stage IV metastatic ocular melanoma metastatic to the liver. The average survival of patients with this type of cancer is approximately 7 months, suggesting that treatment with DSF substantially increased the patient's survival (by > 46 months) [50]. Further studies are needed to determine the effect of DSF in human GBM xenografts.

DSF is clearly a potential anti-cancer drug with the ability to suppress tumor progression through multiple mechanisms. The proteasome inhibitor bortezomib (Velcade) is FDA approved for the treatment of multiple myeloma and mantle cell lymphoma, and is showing positive results in clinical trials of recurrent glioma [15]. However, the main drawbacks of this drug include limited brain penetrance and its overall toxicity in humans. The development of proteasome inhibitors that overcome these limitations, such as DSF, could be of greater clinical significance in the treatment of solid tumors. Here, we show that DSF can inhibit the proliferation of GSCs, suggesting that this drug may have the potential to improve prognosis in GBM patients. These observations, combined with the ability to administer DSF long-term, justify clinical trials of DSF either as a monotherapy or as an adjuvant with other agents.

## FUNDING

We gratefully acknowledge The Ben and Catherine Ivy Foundation for support of this research.

## Supplementary Tables and Figures




