# Supplementary material for: High-Throughput Chemical Screens Identify Disulfiram as an Inhibitor of Human Glioblastoma Stem Cells
Source: Oncotarget. 2012 Oct 23;3(10):1124–36. doi: 10.18632/oncotarget.707 (PMC3717950; doi:10.18632/oncotarget.707)
Supplement: Supplementary file 1 [file oncotarget-03-1124-s001.pdf]

High-Throughput Chemical Screens Identify Disulfiram as an Inhibitor of Human Glioblastoma Stem Cells – Hothi et al

Table S1: Patient samples used in this study.

	Patient	Gender	Age	Diagnosis	Resection location
1	SN143	Male	74	Glioblastoma	Left Temporal
2	SN175	Female	49	Glioblastoma	Left Parietal
3	SN179	Male	50	Glioblastoma	Right Frontal
4	SN186	Male	75	Glioblastoma	Right Temporal
5	SN187	Male	57	Glioblastoma	Right Parietal
6	SN195	Female	59	Glioblastoma	Right Parietal
7	SN206	Male	76	Glioblastoma	Right Frontal
8	SN211	Female	36	Glioblastoma	Right Parietal
9	SN235	Male	73	Glioblastoma	Right Parietal
10	G144	Male	51	Glioblastoma	Unknown

G144 patient information is taken from the corresponding publication [Pollard SM, et al. Cell Stem Cell. 2009; 4(6): 568-580]. The G144 GSC line was obtained from the BioRep Cell Repository (Milan, Italy). All other patients underwent tumor resection at Swedish Medical Center (Seattle, WA).

Table S2. Common inhibitors of GSC proliferation.

Compound		Formula	Mol. weight	Activity	Status
1	10-Hydroxycamptothecin	$C_{20}H_{16}N_2O_5$	364.36	Antineoplastic	Experimental
2	2,3-Dichloro-5,8-dihydroxynapthoquinone	$C_{10}H_4Cl_2O_4$	259.05	Apoptosis inducer	Experimental
3	3β-Hydroxy-23,24-bisnorchol-5-enic acid	$C_{22}H_{34}O_3$	346.51	Undetermined	Experimental
4	4’-Demethylepipodophyllotoxin	$C_{21}H_{20}O_8$	400.39	Antineoplastic	Experimental
5	Acetyl gambogic acid	$C_{40}H_{47}O_9$	671.81	Derivative of gambogic acid, undetermined	Experimental
6	Albendazole	$C_{12}H_{15}N_3O_2S$	265.34	Antihelminthic	USP
7	Amsacrine	$C_{22}H_{20}N_2O_3S$	392.48	Antineoplastic, immune suppressive	USAN, INN, BAN
8	Ancitabine hydrochloride	$C_9H_{12}ClN_3O_4$	261.67	Antineoplastic	INN, JAN
9	Anisomycin	$C_{14}H_{19}NO_4$	265.31	Antibacterial, inhibits protein synthesis	Experimental
10	Anthothecol	$C_{28}H_{32}O_7$	480.56	Undetermined	Experimental
11	Antimycin A	$C_{27}H_{38}N_2O_9$	534.61	Antifungal, antiviral	Experimental
12	Atorvastatin calcium	$C_{33}H_{33}CaFNO_5$	582.71	Antihyperlipidemic, HMGCoA reductase inhibitor	USAN, INN, BAN
13	Benzalkonium chloride	$C_{22}H_{40}ClN$	354.02	Topical anti-infective	USAN, INN, BAN, JAN
14	β-Peltatin	$C_{22}H_{22}O_8$	414.41	Natural product with antitumor activity	Experimental
15	Camptothecin	$C_{20}H_{16}N_2O_4$	348.36	Antineoplastic	Experimental
16	Colchicine	$C_{22}H_{25}NO_6$	399.45	Anti-infective, antimitotic, antigout agent	USP, JAN
17	Convallatoxin	$C_{29}H_{42}O_{10}$	550.65	Cardiotonic	Experimental
18	Cytarabine	$C_9H_{13}N_3O_5$	243.22	Antineoplastic, antiviral	USP, INN, BAN, JAN
19	Dactinomycin	$C_{62}H_{86}N_{12}O_{16}$	1255.45	Antineoplastic, intercalating agent	USP, INN, BAN, JAN
20	Daunorubicin	$C_{27}H_{29}NO_{10}$	527.53	Antineoplastic	USAN, INN, BAN, JAN
21	Deguelin	$C_{23}H_{22}O_6$	394.43	Antineoplastic, antiviral, insecticide	Experimental
22	Deoxysappanone B 7,4-dimethyl ether	$C_{18}H_{18}O_5$	314.34	Undetermined	Experimental

23	Deoxysappanone B 7,3-dimethyl ether acetate	$C_{20}H_{20}O_6$	356.38	Undetermined	Experimental
24	Digitonin	$C_{56}H_{92}O_{29}$	1229.34	Cardiotonic	Experimental
25	Digitoxin	$C_{41}H_{64}O_{13}$	764.96	Cardiotonic, inotropic	USP, INN, BAN, JAN
26	Digoxigenin	$C_{23}H_{34}O_5$	390.52	Aglycon of digitoxin	Experimental
27	Digoxin	$C_{41}H_{64}O_{14}$	780.96	Cardiac stimulant	USP, INN, BAN, JAN
28	Disulfiram	$C_{10}H_{20}N_2S_4$	296.54	Alcohol antagonist	USP, INN, BAN, JAN
29	Doxorubicin	$C_{27}H_{29}NO_{11}$	543.53	Anineoplastic	USAN, INN, BAN
30	Emetine	$C_{29}H_{42}Cl_2N_2O_4$	553.58	Inhibits RNA, DNA and protein synthesis	USP, BAN
31	Epirubicin hydrochloride	$C_{27}H_30ClNO_{11}$	579.99	Antineoplastic	USAN, INN, BAN, JAN
32	Etoposide	$C_{29}H_{32}O_{13}$	588.57	Antineoplastic	USP, INN, BAN, JAN
33	Fenbendazole	$C_{15}H_{13}N_3O_2S$	299.35	Antihelminthic	USAN, INN, BAN
34	Fludarabine phosphate	$C_{10}H_{13}FN_5O_7P$	365.22	Antineoplastic	USP, INN, BAN
35	Fluvastatin	$C_{24}H_{26}FNO_4$	411.48	Antihyperlipidemic, HMGCoA reductase inhibitor	USAN, INN, BAN
36	Gambogic acid	$C_{38}H_{44}O_8$	628.77	Antiinflammatory, apoptosis inducer	Experimental
37	Gitoxigenin diacetate	$C_{27}H_{38}O_7$	474.6	Aglycon of gitoxin	Experimental
38	Gitoxin	$C_{41}H_{64}O_{14}$	780.96	Cardiotonic	Experimental
39	Gramicidin	$C_{60}H_{92}N_{12}O_{10}$	1141.48	Antibacterial	USP
40	Homidium bromide	$C_{21}H_{20}BrN_3$	394.32	Antiprotozoal, intercalcate with DNA	INN, BAN
41	Mebendazole	$C_{16}H_{13}N_3O_3$	295.3	Antihelminthic	USP, INN, BAN, JAN
42	Methotrexate	$C_{20}H_{22}N_8O_5$	454.45	Antineoplastic, antirheumatic	USP, INN, BAN, JAN
43	Methylene blue	$C_{16}H_{20}ClN_3OS$	337.87	Antimethemoglobinemic, cyanide antidote	USP, INN, BAN
44	Mitomycin C	$C_{15}H_{18}N_4O_5$	334.33	Antineoplastic	USP, INN, BAN, JAN
45	Mitoxantrone hydrochloride	$C_{22}H_{30}Cl_2N_4O_6$	517.41	Antineoplastic	USP, INN, BAN, JAN
46	Monensin sodium	$C_{37}H_{63}NaO_{10}$	690.9	Antibacterial	USP, INN, BAN
47	Niclosamide	$C_{13}H_8Cl_2N_2O_4$	327.13	Antihelminthic, teniacide	USAN, INN, BAN
48	Oxibendazole	$C_{12}H_{15}N_3O_3$	249.27	Antihelminthic	USAN, INN, BAN
49	Oubain	$C_{29}H_{44}O_{12}$	584.67	Cardiotonic, antiarrhythmic, hypertensive	JAN
50	Paclitaxel	$C_{47}H_{51}NO_{14}$	853.93	Antineoplastic	USP, INN, BAN
51	Pararosaniline pamoate	$C_{42}H_{33}N_3O_6$	675.75	Antihelminthic, antischistosomal	USAN, INN
52	Patulin	$C_7H_6O_4$	154.12	Antibacterial	Experimental
53	Peruvoside	$C_{30}H_{44}O_9$	548.68	Cardiotonic	Experimental
54	Phenethyl caffeate (CAPE)	$C_{17}H_{16}O_4$	284.31	Antineoplastic, antiinflammatory	Experimental
55	Picropodophyllin	$C_{22}H_{22}O_8$	414.42	Antineoplastic, insulin growth factor receptor inhibitor	Experimental
56	Plumbagin	$C_{11}H_8O_3$	188.18	Antibacterial, antifungal, tuberculostatic	Experimental
57	Podofilox	$C_{22}H_{22}O_8$	414.42	Antineoplastic, inhibits microtubule assembly and DNA	USAN, BAN

58	Podophyllin acetate	$C_{24}H_{24}O_9$	456.45	Undetermined	Experimental
59	Pristimerin	$C_{30}H_{40}O_4$	464.65	Antineoplastic, antiinflammatory	Experimental
60	Puromycin hydrochloride	$C_{22}H_{31}Cl_2N_7O_5$	544.44	Antineoplastic, antiprotozoal	USAN, INN, BAN
61	Pyrithione zinc	$C_{10}H_{10}N_2O_2S_2Z$	319.70	Antibacterial, antifungal, antiseborrheic	USAN, INN, BAN
62	Pyrvinium pamoate	$C_{49}H_{43}N_3O_6$	769.91	Antihelminthic	USP, BAN, JAN
63	Rotenone	$C_{23}H_{22}O6$	394.43	Acaricide, ectoparasiticide, antineoplastic	Agricultural use
64	Salinomycin, sodium	$C_{42}H_{69}NaO_{11}$	773.00	Antibacterial	INN, BAN
65	Simvastatin	C25H38O5	418.58	Antihyperlipidemic, HMGCoA reductase inhibitor	USP, INN, BAN
66	Sodium tetradecyl sulfate	$C_{14}H_{29}NaO_4S$	316.44	Sclerosing agent	INN
67	Strophanthidin	$C_{23}H_{32}O_6$	404.51	Cardiotonic	Experimental
68	Strophanthidinic acid lactone acetate	$C_{25}H_{32}O_7$	444.53	Undetermined	Experimental
69	Teniposide	$C_{32}H_{32}O_{13}S$	656.67	Antineoplastic	USAN, INN, BAN
70	Tetrachloroisophthalonitrile	$C_8Cl_4N_2$	265.91	Antifungal	Experimental
71	Thimerosal	$C_9H_9HgNaO_2S$	404.81	Anti-infective, preservative	USP, INN, BAN, JAN
72	Thiram	$C_6H_{12}N_2S_4$	240.43	Antifungal	USAN, INN
73	Topotecan hydrochloride	$C_{23}H_{24}ClN_3O_5$	457.92	Antineoplastic, topoisomerase I inhibitor	USAN, INN, BAN
74	Trifluoperazine hydrochloride	$C_{21}H_{26}Cl_2F_3N_3S$	480.43	Antipsychotic	USP, INN, BAN, JAN
75	Tyrothricin	$C_{66}H_{85}N_{11}O_{15}$	1272.48	Topical antibacterial	USP, INN, BAN
76	Valinomycin	$C_{54}H_{90}N_6O_{18}$	1111.35	Antibacterial	Experimental
77	Vinblastine sulfate	$C_{46}H_{60}N_4O_{13}S$	909.07	Antineoplastic, spindle poison	USP, INN, BAN, JAN
78	Vincristine sulfate	$C_{46}H_{58}N_4O_{14}S$	923.06	Antineoplastic	USP, INN, BAN, JAN

Abbreviations:
BAN: British Approved Name
INN: International Nonproprietary Name
JAN: Japanese Accepted Name
USAN: United States Adopted Name
USP: United States Pharmacopeia

Table S3. Specific inhibitors of GSC proliferation.

Compound		Formula	Mol. weight	Activity	Active against				
					SN143	SN175	SN179	SN186	G144
1	3,4-Dimethoxyflavone	$C_{17}H_{14}O_4$	282.3	Undetermined	X	X			X
2	3β-Acetoxydeoxodihydrogedunin	$C_{30}H_{40}O_8$	528.65	Undetermined	X				
3	3-Methoxycatechol	$C_7H_8O_3$	140.14	Undetermined		X			X
4	4'-Hydroxychalcone	$C_{15}H_{12}O_2$	224.26	Undetermined		X			
5	5α-Cholestan-3β-ol-6-one	$C_{27}H_{48}$	372.68	Undetermined			X	X	
6	7-Oxocholesterol	$C_{27}H_{44}O_2$	400.65	Undetermined	X			X	
7	Acexamic acid	$C_8H_{15}NO_3$	173.21	Wound healing agent, undetermined			X		
8	Adenosine	$C_{10}H_{13}N_5O_4$	267.25	Antiarrhythmic			X	X	
9	Adenosine phosphate	$C_{10}H_{14}N_5O_7P$	347.23	Vasodilator, neuromodulator			X	X	
10	Alexidine hydrochloride	$C_{26}H_{58}C_{l2}N_{10}$	581.73	Antibacterial				X	
11	Bleomycin	$C_{55}H_{84}N_{17}O_{21}S_3$	1415.55	Antineoplastic				X	
12	Brompheniramine maleate	$C_{20}H_{23}BrN_2O_4$	435.32	H1 antihistamine	X	X			
13	Canthaxanthin	$C_{40}H_{52}O_2$	564.86	Undetermined	X				X
14	Carboplatin	$C_6H_{12}N_2O_4Pt$	371.26	Antineoplastic	X				
15	Carmofur	$C_{11}H_{16}FN_3O_3$	257.27	Antineoplastic	X	X			
16	Cedrelone	$C_{26}H_{30}O_5$	422.53	Undetermined	X	X	X		
17	Cetrimonium bromide	$C_{19}H_{42}BrN$	364.45	Component of antiseptic, undetermined		X			
18	Clofazimine	$C_{27}H_{22}C_{l2}N_4$	473.41	Antibacterial			X		
19	Clonidine hydrochloride	$C_9H_{10}C_{l3}N_3$	266.56	Antihypertensive	X	X	X		
20	Cryptotanshinone	$C_{19}H_{20}O_3$	296.37	Inhibits angiogenesis, undetermined		X		X	
21	Deoxyadenosine	$C_{10}H_{13}N_5O_3$	251.25	Undetermined			X	X	
22	Diallyl trisulfide	$C_6H_{10}S_3$	178.28	Undetermined			X		
23	Ethacrynic acid	$C_{13}H_{12}C_{l2}O_4$	303.14	Diuretic			X		
24	Flourouracil	$C_4H_3FN_2O_2$	130.08	Antineoplastic		X			
25	Fluphenazine hydrochloride	$C_{22}H_{28}C_{l2}F_3N_3OS$	510.45	H1 antihistamine			X		X
26	Gentian violet	$C_{25}H_{30}ClN_3$	407.99	Antibacterial	X				
27	Gossypol	$C_{30}H_{30}O_8$	518.57	Antineoplastic		X			
28	Harmaline	$C_{13}H_{14}N_2O$	214.25	Psychotropic, CNS stimulant, antiparkinsons		X	X		
29	Harmine	$C_{13}H_{12}N_2O$	212.25	Psychotropic, CNS stimulant, antiparkinsons		X			

30	Helenine	$C_{15}H_{20}O_2$	232.33	Antihelminthic		X	X		X
31	Hexetidine	$C_{21}H_{45}N_3$	339.61	Antifungal	X		X	X	
32	Irigenin	$C_{18}H_{16}O_8$	360.32	Undetermined	X	X			
33	Lappaconitine	$C_{32}H_{44}N_2O_8$	584.72	Antiarrhythmic		X	X		X
34	Melphalan	$C_{13}H_{18}Cl_2N_2O_2$	305.21	Antineoplastic		X	X		
35	Methylbenzethonium chloride	$C_{28}H_{44}ClNO_2$	462.13	Anti-infective			X	X	
36	Mevastatin	$C_{23}H_{34}O_5$	390.52	Antihyperlipidermic			X	X	
37	Minoxidil	$C_9H_{15}N_5O$	209.25	Antihypertensive		X			
38	Mycophenolate mofetil	$C_{23}H_{31}NO_7$	433.49	Immunosuppressant		X	X		
39	Nadide	$C_{21}H_{27}N_7O_{14}P_2$	663.44	Alcohol antagonist			X		
40	Nithiamide	$C_5H_5N_3O_3S$	187.18	Antibacterial		X			
41	Norcantharidin	$C_8H_8O_4$	168.15	Antineoplastic, protein phosphatase inhibitor			X		
42	Obtusaquinone	$C_{16}H_{14}O_3$	254.29	Undetermined		X			X
43	Osajin	$C_{25}H_{24}O_5$	404.46	Apoptosis inducer, undetermined	X				
44	Oxelaidin citrate	$C_{26}H_{41}NO_{10}$	527.62	Antitussive, undetermined			X		
45	Pangamic acid sodium	$C_{10}H_{18}NNaO_8$	303.25	Undetermined		X			X
46	Pindolol	$C_{14}H_{20}N_2O_2$	248.33	Antihypertensive		X	X	X	
47	Piplartine	$C_{17}H_{19}NO_5$	317.34	Antibronchitis, undetermined		X	X	X	
48	Pramoxine hydrochloride	$C_{17}H_{28}ClNO_3$	329.87	Anesthetic, undetermined			X		
49	Pyrromycin	$C_{30}H_{35}NO_{11}$	585.61	Antibacterial		X			
50	Rosuvastatin calcium	$C_{22}H_{27}CaFN_3O_6S$	520.62	Antihyperlipidermic			X		
51	Securinine	$C_{13}H_{15}NO_2$	217.27	Psychotropic, CNS stimulant		X			
52	Sirolimus	$C_{51}H_{79}NO_{13}$	914.17	Immunosuppressant, antineoplastic	X	X			
53	Sulconazole nitrate	$C_{18}H_{16}C_{l3}N_3O_3S$	460.77	Antifungal			X		X
54	Suloctidil	$C_{20}H_{35}NO_S$	337.57	Peripheral vasodilator			X	X	X
55	Trimebutine maleate	$C_{26}H_{33}NO_9$	503.55	Antispasmodic, undetermined			X		
	Total active agents				14	27	29	14	10

Table S4. Half maximal inhibitory concentrations (IC_{50}) for common inhibitors of GSC proliferation.

Compound		NSC	SN143	SN175	SN179	SN186	G144	Average	SD
1	10-Hydroxycamptothecin	0.177	0.004	0.187	0.169	0.01	0.005	0.075	0.095
2	2,3-Dichloro-5,8-dihydroxynapthoquinone	0.812	0.131	0.167	0.198	0.184	0.173	0.171	0.025
3	3β-Hydroxy-23,24-bisnorchol-5-enic acid	0.324	0.330	0.542	0.375	0.122	0.310	0.336	0.150
4	4’-Demethylepipodophyllotoxin	0.151	0.039	0.077	0.029	0.023	0.022	0.038	0.023
5	Acetyl gambogic acid	0.920	0.203	0.203	0.603	0.425	0.476	0.382	0.176
6	Albendazole	0.388	0.367	0.427	0.363	0.370	0.903	0.486	0.235
7	Amsacrine	0.072	0.174	0.132	0.678	0.077	0.195	0.251	0.243
8	Ancitabine hydrochloride	0.191	0.018	0.218	0.093	0.208	0.549	0.217	0.203
9	Anisomycin	0.129	0.034	0.062	0.100	0.034	0.056	0.057	0.027
10	Anthothecol	0.870	0.234	0.273	0.579	0.396	0.250	0.346	0.145
11	Antimycin A	0.084	0.389	0.004	3.90	0.012	3.80	1.621	2.041
12	Atorvastatin calcium*	54.7%	26.3%	46.2%	12.1%	21.93%	31.2%	27.55%	12.58
13	Benzalkonium chloride	5.073	5.142	3.771	3.669	2.758	1.10	3.288	1.49
14	β-Peltatin	0.127	0.057	0.082	0.048	0.041	0.035	0.053	0.018
15	Camptothecin	0.148	0.302	0.630	0.267	0.010	0.420	0.326	0.226
16	Colchicine	0.334	0.017	0.016	0.014	0.0014	0.003	0.010	0.0075
17	Convallatoxin*	117.6%	9.5E-05	0.053	0.023	0.023	0.0001	0.020	0.0218
18	Cytarabine	0.515	0.042	0.327	0.250	0.852	3.50	0.994	1.432
19	Dactinomycin	0.115	0.0033	0.0038	0.0009	0.025	0.003	0.007	0.01
20	Daunorubicin	0.267	0.077	0.239	0.202	0.051	0.159	0.146	0.08
21	Deguelin*	0.021	0.10	0.201	83.2%	0.085	1.05	0.359	0.464
22	Deoxysappanone B 7,4’-dimethyl ether	1.507	0.188	0.291	0.189	0.178	0.224	0.214	0.046
23	Deoxysappanone B 7,3-dimethyl ether acetate*	1.32	28.6%	28.6%	39.5%	36.24%	38.3%	34.25%	5.29
24	Digitonin	1.706	0.798	0.471	0.579	0.421	0.894	0.633	0.206
25	Digitoxin*	95.1%	0.106	0.307	0.166	0.149	0.094	0.164	0.085
26	Digoxigenin*	120.8%	0.466	1.818	0.415	0.24	0.263	0.640	0.665
27	Digoxin*	103.5%	0.059	0.261	0.107	0.074	0.028	0.106	0.0913
28	Disulfiram	0.283	0.028	0.037	0.04	0.028	0.039	0.034	0.0059

29	Doxorubicin	0.144	0.045	0.129	0.169	0.037	0.067	0.089	0.0573
30	Emetine	0.112	0.031	0.040	0.038	0.064	0.041	0.043	0.0125
31	Epirubicin hydrochloride	1.20	0.169	0.387	0.227	0.048	0.737	0.313	0.2663
32	Etoposide	6.89	1.30	0.447	5.62	0.28	1.10	1.749	2.206
33	Fenbendazole	1.918	0.836	1.124	0.661	0.562	0.892	0.815	0.2177
34	Fludarabine phosphate*	121.5%	98.4%	0.135	0.143	0.112	0.109	0.125	0.0168
35	Fluvastatin	2.32	14.69	18.82	0.552	0.386	2.0	7.290	8.786
36	Gambogic acid	0.31	0.076	0.109	0.268	0.0997	0.115	0.134	0.0766
37	Gitoxigenin diacetate	5.027	0.096	0.289	0.171	0.096	0.049	0.140	0.094
38	Gitoxin*	122.3%	0.276	1.693	0.384	0.238	0.097	0.538	0.654
39	Gramicidin	0.918	0.001	0.0001	0.031	0.018	0.00002	0.010	0.014
40	Homidium bromide*	9.1%	5.3%	4.8%	1.7%	30.45%	15.8%	11.61%	11.8
41	Mebendazole	0.458	0.339	0.546	0.375	0.282	0.335	0.375	0.101
42	Methotrexate*	0.162	86.7%	71.02%	0.128	0.061	0.293	0.161	0.1194
43	Methylene blue*	11.3%	3.7%	1.9%	7.5%	5.83%	3.9%	4.57%	2.15
44	Mitomycin C	9.199	0.0695	0.425	0.818	0.110	0.268	0.338	0.303
45	Mitoxantrone hydrochloride	0.208	0.605	0.136	0.125	0.321	0.052	0.248	0.223
46	Monensin sodium	0.896	0.256	0.33	0.085	0.073	0.234	0.196	0.1123
47	Niclosamide	2.466	1.43	1.372	1.194	1.568	1.20	1.353	0.16
48	Oxibendazole	1.371	0.416	0.822	0.687	0.489	0.53	0.589	0.164
49	Ouabain	4.20	0.005	0.134	0.031	0.006	0.00013	0.035	0.0565
50	Paclitaxel*	0.126	0.013	42.3%	56.9%	0.0028	0.009	0.008	0.005
51	Pararosaniline pamoate*	9.7%	1.1%	1.6%	1.2%	1.3%	1.1%	1.26%	0.21
52	Patulin	0.383	0.145	0.282	0.164	0.174	0.290	0.211	0.069
53	Peruvoside*	119.6%	1.5E-05	0.1196	0.015	0.012	0.006	0.031	0.05
54	Phenethyl caffeate (CAPE)	13.25	1.125	0.768	0.341	0.802	0.941	0.796	0.29
55	Picropodophyllin	0.991	0.291	0.44	0.371	0.39	0.148	0.328	0.114
56	Plumbagin	0.865	0.693	1.068	0.845	0.443	0.228	0.655	0.33
57	Podofilox	0.028	0.017	0.038	0.06	0.007	0.0008	0.0243	0.0243
58	Podophyllin acetate*	17.4%	0.008	0.029	0.004	0.0354	0.121	0.0395	0.0475
59	Pristimerin	1.577	0.489	0.456	1.019	0.516	0.436	0.583	0.246
60	Puromycin hydrochloride*	104.6%	0.206	0.365	0.410	0.129	0.194	0.2609	0.1204

61	Pyrithione zinc*	1.79%	0.2%	0.2%	0.3%	0.65%	0.3%	0.33%	0.19
62	Pyrvinium pamoate	0.871	0.08	0.149	0.187	0.124	0.26	0.1598	0.068
63	Rotenone*	54.2%	20.7%	24.8%	47.7%	49.28%	41.9%	36.88%	13.26
64	Salinomycin, sodium	0.143	0.293	0.508	0.226	0.240	0.590	0.371	0.167
65	Simvastatin	2.16	1.97	2.36	0.133	0.362	1.70	1.305	0.997
66	Sodium tetradecyl sulfate*	31.9%	38.6%	31.9%	29.7%	27.48%	33.0%	32.14%	4.19
67	Strophanthidin*	124.7%	0.145	0.844	0.264	0.294	0.082	0.326	0.3023
68	Strophanthidinic acid lactone acetate*	110.8%	0.266	0.970	0.623	0.286	0.124	0.4538	0.3418
69	Teniposide	0.12	0.036	1.054	0.195	0.116	0.054	0.291	0.431
70	Tetrachloroisophthalonitrile	0.061	0.012	0.035	0.042	0.036	0.026	0.0302	0.0117
71	Thimerosal*	0.4%	0.1%	0.1%	0.1%	0.009%	0.2%	0.1%	0.07
72	Thiram	0.317	0.044	0.042	0.046	0.029	0.048	0.0419	0.0075
73	Topotecan hydrochloride	0.125	0.038	0.05	1.737	0.043	0.013	0.376	0.7608
74	Trifluoperazine hydrochloride*	106.4%	102.4%	103.3%	0.578	0.303	1.70	0.860	0.74
75	Tyrothricin*	0.73%	0.2%	0%	0.5%	0.4%	0.3%	0.28%	0.19
76	Valinomycin	0.0195	0.001	0.035	0.016	0.008	0.007	0.0135	0.0132
77	Vinblastine sulfate	0.103	0.002	0.0018	0.011	0.001	0.003	0.004	0.0041
78	Vincristine sulfate	0.085	0.04	1.2E-07	0.028	0.003	4E-08	0.0142	0.0186

IC_{50} values (μM) for GSCs are presented with the average and standard deviation (SD).

*Data are presented as % survival at 10 μM where no clear sigmodial relationship was observed in the tested concentration range (0.01 – 10 μM).

Table S5. Polar surface area (PSA) for inhibitors of GSC proliferation.

Compound		IC_{50} (μM)	Activity	Status	Violations	PSA (Å^2)
1	10-Hydroxycamptothecin	0.075	Antineoplastic	Experimental	0	77.96
2	2,3-Dichloro-5,8-dihydroxynapthoquinone	0.171	Apoptosis inducer	Experimental	0	74.6
3	3β-Hydroxy-23,24-bisnorchol-5-enic acid	0.336	Undetermined	Experimental	0	35.53
4	4'-Demethylepipodophyllotoxin	0.038	Antineoplastic	Experimental	0	81.68
5	Acetyl gambogic acid	0.382	Derivative of gambogic acid, undetermined	Experimental	2	159.36
6	Albendazole	0.486	Antihelminthic	USP	0	72.66
7	Amsacrine	0.251	Antineoplastic, immune suppressive	USAN, INN, BAN	0	71.12
8	Ancitabine hydrochloride	0.217	Antineoplastic	INN, JAN	0	98.37
9	Anisomycin	0.057	Antibacterial, inhibits protein synthesis	Experimental	0	48.0
10	Anthothecol	0.346	Undetermined	Experimental	1	106.34
11	Antimycin A	1.621	Antifungal, antiviral	Experimental	2	128.75
12	Atorvastatin calcium	27.55%	Antihyperlipidemic, HMGCoA reductase inhibitor	USAN, INN, BAN	2	80.59
13	Benzalkonium chloride	3.288	Topical anti-infective	USAN, INN, BAN, JAN	0	0
14	β-Peltatin	0.053	Natural product with antitumor activity	Experimental	0	81.63
15	Camptothecin	0.326	Antineoplastic	Experimental	0	79.93
16	Colchicine	0.010	Anti-infective, antimitotic, antigout agent	USP, JAN	0	74.3
17	Convallatoxin	0.020	Cardiotonic	Experimental	3	107.98
18	Cytarabine	0.994	Antineoplastic, antiviral	USP, INN, BAN, JAN	1	72.83
19	Dactinomycin	0.007	Antineoplastic, intercalating agent	USP, INN, BAN, JAN	3	355.54
20	Daunorubicin	0.146	Antineoplastic	USAN, INN, BAN, JAN	3	185.84
21	Deguelin	0.359	Antineoplastic, antiviral, insecticide	Experimental	0	63.22
22	Deoxysappanone B 7,4'-dimethyl ether	0.214	Undetermined	Experimental	0	53.99
23	Deoxysappanone B 7,3-dimethyl ether acetate	34.25%	Undetermined	Experimental	0	71.06
24	Digitonin	0.633	Cardiotonic	Experimental	3	267.67
25	Digitoxin	0.164	Cardiotonic, inotropic	USP, INN, BAN, JAN	3	182.83
26	Digoxigenin	0.640	Aglycon of digitoxin	Experimental	0	53.99
27	Digoxin	0.106	Cardiac stimulant	USP, INN, BAN, JAN	3	137.06
28	Disulfiram*	0.034	Alcohol antagonist	USP, INN, BAN, JAN	0	121.26
29	Doxorubicin	0.089	Anineoplastic	USAN, INN, BAN	3	206.07
30	Emetine	0.043	Inhibits RNA, DNA and protein synthesis	USP, BAN	0	43.4
31	Epirubicin hydrochloride	0.313	Antineoplastic	USAN, INN, BAN, JAN	3	128.29

32	Etoposide	1.749	Antineoplastic	USP, INN, BAN, JAN	2	160.83
33	Fenbendazole	0.815	Antihelminthic	USAN, INN, BAN	0	72.66
34	Fludarabine phosphate	0.125	Antineoplastic	USP, INN, BAN	2	195.88
35	Fluvastatin	7.290	Antihyperlipidemic, HMGCoA reductase inhibitor	USAN, INN, BAN	0	49.69
36	Gambogic acid	0.134	Antiinflammatory, apoptosis inducer	Experimental	2	119.36
37	Gitoxigenin diacetate	0.140	Aglycon of gitoxin	Experimental	0	53.99
38	Gitoxin	0.538	Cardiotonic	Experimental	3	137.06
39	Gramicidin	0.010	Antibacterial	USP	3	209.58
40	Homidium bromide	11.61%	Antiprotozoal, intercalcate with DNA	INN, BAN	0	55.92
41	Mebendazole	0.375	Antihelminthic	USP, INN, BAN, JAN	0	64.43
42	Methotrexate	0.161	Antineoplastic, antirheumatic	USP, INN, BAN, JAN	2	134.19
43	Methylene blue	4.57%	Antimethemoglobinemic, cyanide antidote	USP, INN, BAN	0	43.91
44	Mitomycin C	0.338	Antineoplastic	USP, INN, BAN, JAN	1	146.89
45	Mitoxantrone hydrochloride	0.248	Antineoplastic	USP, INN, BAN, JAN	2	84.02
46	Monensin sodium	0.196	Antibacterial	USP, INN, BAN	2	120.37
47	Niclosamide	1.353	Antihelminthic, teniacide	USAN, INN, BAN	1	75.36
48	Oxibendazole	0.589	Antihelminthic	USAN, INN, BAN	0	56.59
49	Ouabain	0.035	Cardiotonic, antiarrhythmic, hypertensive	JAN	3	118.6
50	Paclitaxel	0.008	Antineoplastic	USP, INN, BAN	2	221.29
51	Pararosaniline pamoate	1.26%	Antihelminthic, antischistosomal	USAN, INN	2	71.06
52	Patulin	0.211	Antibacterial	Experimental	0	44.76
53	Peruvoside	0.031	Cardiotonic	Experimental	1	131.75
54	Phenethyl caffeate (CAPE)	0.796	Antineoplastic, antiinflammatory	Experimental	0	44.76
55	Picropodophyllin	0.328	Antineoplastic, insulin growth factor receptor inhibitor	Experimental	0	81.68
56	Plumbagin	0.655	Antibacterial, antifungal, tuberculostatic	Experimental	0	43.37
57	Podofilox	0.0243	Antineoplastic, inhibits microtubule assembly & DNA	USAN, BAN	0	92.68
58	Podophyllin acetate	0.0395	Undetermined	Experimental	1	151.69
59	Pristimerin	0.583	Antineoplastic, antiinflammatory	Experimental	1	52.6
60	Puromycin hydrochloride	0.2609	Antineoplastic, antiprotozoal	USAN, INN, BAN	2	107.31
61	Pyrithione zinc	0.33%	Antibacterial, antifungal, antiseborrheic	USAN, INN, BAN	0	55.56
62	Pyrvinium pamoate	0.1598	Antihelminthic	USP, BAN, JAN	1	127.0
63	Rotenone	36.88%	Acaricide, ectoparasiticide, antineoplastic	Agricultural use	0	63.22
64	Salinomycin, sodium	0.371	Antibacterial	INN, BAN	3	117.21
65	Simvastatin	1.305	Antihyperlipidemic, HMGCoA reductase inhibitor	USP, INN, BAN	0	61.83
66	Sodium tetradecyl sulfate	32.14%	Sclerosing agent	INN	1	71.98

67	Strophanthidin	0.326	Cardiotonic	Experimental	0	71.06
68	Strophanthidinic acid lactone acetate	0.4538	Undetermined	Experimental	0	88.13
69	Teniposide	0.291	Antineoplastic	USAN, INN, BAN	2	156.07
70	Tetrachloroisophthalonitrile	0.0302	Antifungal	Experimental	0	47.58
71	Thimerosal	0.1%	Anti-infective, preservative	USP, INN, BAN, JAN	0	65.43
72	Thiram	0.0419	Antifungal	USAN, INN	0	121.26
73	Topotecan hydrochloride	0.376	Antineoplastic, topoisomerase I inhibitor	USAN, INN, BAN	0	81.2
74	Trifluoperazine hydrochloride	0.86	Antipsychotic	USP, INN, BAN, JAN	1	35.02
75	Tyrothricin	0.28%	Topical antibacterial	USP, INN, BAN	3	268.17
76	Valinomycin	0.0135	Antibacterial	Experimental	4	332.4
77	Vinblastine sulfate	0.004	Antineoplastic, spindle poison	USP, INN, BAN, JAN	2	121.24
78	Vincristine sulfate	0.0142	Antineoplastic	USP, INN, BAN, JAN	3	254.15

Average IC_{50} (or % survival at 10 μM), violations of the Rue of 5 and PSA are shown. Parameters are taken from the online chemical database ChemSpider. In general, orally administered CNS-active drugs have a PSA below 120 $Å^2$ and those that readily penetrate the BBB have a PSA below 70 $Å^2$ [Kelder J, et al. Pharm Res. 1999; 16: 1514-1519].

*DSF is rapidly converted in the bloodstream to its active metabolite diethyldithiocarbamate (DTTC) or methyl diethylcarbamodithioate (Me-DTTC) [Johansson B. Acta Psychiatr Scand Suppl. 1992; 369: 15-26]. PSA values are 35.33 and 60.63 $Å^2$ for DTTC and Me-DTTC, respectively.

Table S6. Half maximal inhibitory concentrations (IC_{50}) for DSF–Cu against multiple patient-derived GSCs.

**GSC**		**IC_{50} (nM)**
1	SN143	27.5
2	SN175	36.5
3	SN179	39.7
4	SN186	27.6
5	SN187	25.6
6	SN195	56.3
7	SN206	14.5
8	SN211	31.8
9	SN235	12.1
10	G144	39.0
	**Average**	31.1
