# Supplementary material for: High-Throughput Chemical Screens Identify Disulfiram as an Inhibitor of Human Glioblastoma Stem Cells
Source: Oncotarget. 2012 Oct 23;3(10):1124–36. doi: 10.18632/oncotarget.707 (PMC3717950; doi:10.18632/oncotarget.707)
Supplement: Supplementary file 2 [file oncotarget-03-1124-s002.pdf]

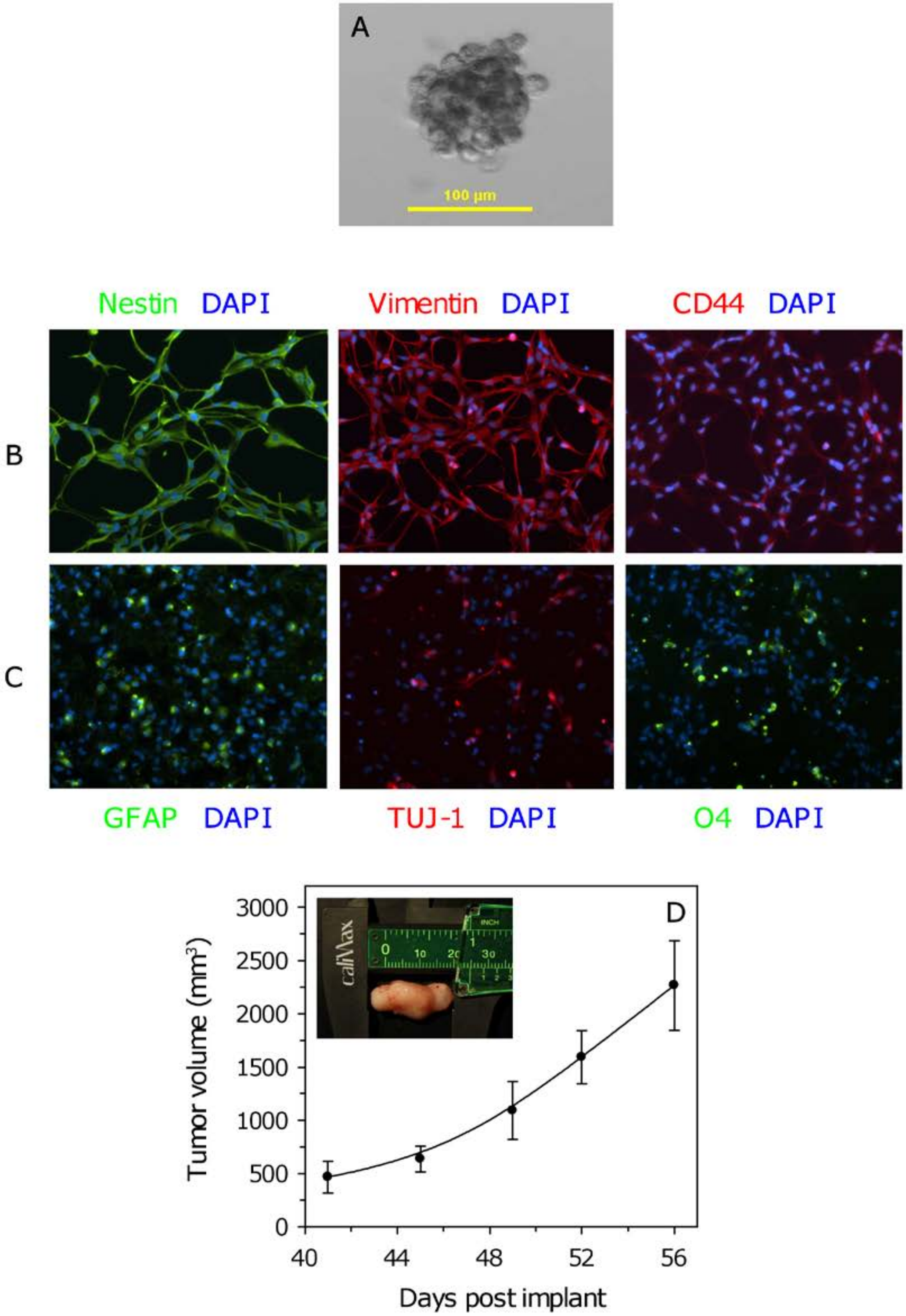

Figure S1. Initial characterization of patient-derived GSCs. (A) Typical example of a neurosphere established from single cells grown in ultra low attachment plates in NSA medium. (B) Immunostaining for the neural progenitor markers nestin, vimentin and CD44. (C)

Immunostaining for the differentiation markers GFAP (astrocytes), TUJ-1 (neurons) and O4 (oligodendrocytes). Cells were grown in NSA medium without growth factors (EGF and FGF-2) for 10 days prior to staining. Parallel cultures grown in the presence of growth factors showed negligible staining for differentiation markers (data not shown). (D) Average tumor volume (n = 5) in immunocompromised mice approximately 6 weeks after implantation of GSCs (1 x 10^6) at passage 5. Inset: Example of tumor mass removed from host. Data in panels A-D correspond to the SN186 GSC line.

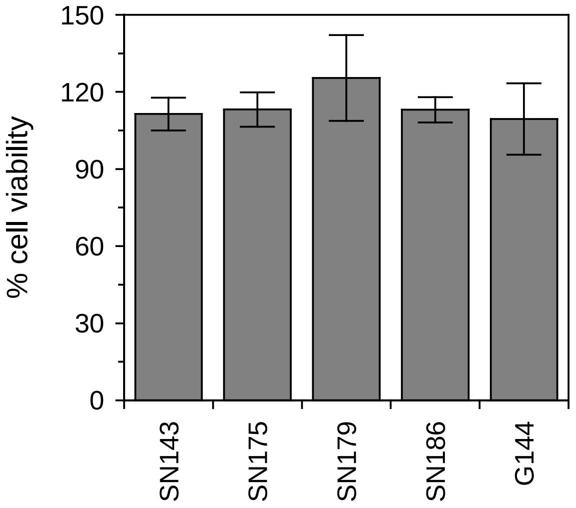

Figure S2: The effect of chloramphenicol on GSC proliferation. Cells were incubated with 10 μM chloramphenicol for 96 hours followed by measurements of cell viability using the CellTiter-Glo luminescent assay. Columns and error bars represent the mean and SD from duplicate measurements, respectively. Chloramphenicol had no affect on GSCs derived from five different GBM patients.
